# Association between intraprocedural oxygen administration and postprocedural pneumothorax after ct-guided transthoracic lung biopsy

**DOI:** 10.1186/s12890-026-04774-2

**Published:** 2026-10-06

**Authors:** Burcu Ozdemir, Deniz Sonmez

**Affiliations:** Department of Radiology, Marmara University Pendik Training and Research Hospital, Istanbul, Türkiye

**Keywords:** Computed tomography, Transthoracic lung biopsy, Pneumothorax, Oxygen administration, Pulmonary hemorrhage

## Abstract

**Background:**

Pneumothorax is the most common complication of computed tomography (CT)-guided transthoracic lung biopsy (TLB). Whether supplemental oxygen administered during the procedure before pneumothorax detection is associated with postprocedural pneumothorax remains unclear. This study evaluated the association between documented intraprocedural oxygen administration and postprocedural pneumothorax.

**Methods:**

This single-center retrospective cohort study included 442 consecutive patients who underwent CT-guided TLB between January 2022 and June 2025. Patients were grouped according to documented oxygen administration during the biopsy and before pneumothorax detection. Oxygen administration was non-standardized, and the exact dose and timing could not be reliably determined. Clinical, imaging, and procedural variables were evaluated. The primary outcome was postprocedural pneumothorax. Multivariable logistic regression analysis was performed to identify factors independently associated with pneumothorax.

**Results:**

Intraprocedural oxygen administration was documented in 118 patients (26.7%), whereas 324 patients (73.3%) received no intraprocedural oxygen. Pneumothorax occurred in 141 patients (31.9%). The pneumothorax rate did not differ significantly between the oxygen and no-oxygen groups [36/118 (30.5%) vs. 105/324 (32.4%); absolute risk difference, − 1.9% points; 95% CI, − 11.6 to 7.8; *p* = 0.705]. No statistically significant association was identified between documented intraprocedural oxygen administration and postprocedural pneumothorax in the univariable analysis (OR, 0.916; 95% CI, 0.581–1.444; *p* = 0.705) or after multivariable adjustment (adjusted OR, 0.807; 95% CI, 0.492–1.323; *p* = 0.395). Non-pleural-based lesion location and CT-detected emphysema were independently associated with pneumothorax, whereas the association with lower subcutaneous tissue thickness should be considered exploratory. Pulmonary hemorrhage and pneumothorax-related intervention rates did not differ significantly between the groups.

**Conclusion:**

In this retrospective cohort, no statistically significant association was identified between documented intraprocedural oxygen administration and postprocedural pneumothorax after CT-guided TLB. This finding does not exclude a preventive effect, which should be evaluated in prospective studies using standardized oxygen protocols.

**Clinical trial number:**

Not applicable

## Background

Computed tomography (CT)-guided transthoracic lung biopsy (TLB) is a minimally invasive procedure widely used for the histopathological diagnosis of pulmonary lesions [1, 2]. Despite its diagnostic value, the procedure carries a risk of complications, most commonly pneumothorax [3, 4].

The risk of pneumothorax after CT-guided lung biopsy is influenced by patient, lesion, and procedural factors. Previous studies have identified emphysema, smaller lesions, lack of pleural contact, a longer needle path through aerated lung, a more oblique needle entry angle, repeated pleural puncture, and patient position as potential risk factors [4–7]. Preventive measures include careful planning of the biopsy route, keeping the needle path through aerated lung as short as possible, and avoiding repeated pleural punctures [2, 8]. Biopsy-tract sealing has also been investigated as a strategy to reduce the risk of pneumothorax [9].

In routine clinical practice, supplemental oxygen may be given during CT-guided lung biopsy according to the patient’s oxygenation status, respiratory symptoms, or ability to tolerate the procedure. Oxygen has also been used to promote the absorption of pleural air after pneumothorax has developed, although this represents a therapeutic rather than a preventive effect [10, 11]. Oxygen may accelerate pleural-air resorption through the nitrogen-washout effect by increasing the diffusion of nitrogen from the pleural space into the blood [11]. Previous fluoroscopy-guided studies of 100% oxygen during lung biopsy reported inconsistent results. One study found fewer and smaller pneumothoraces [12], whereas the other found no statistically significant reduction in pneumothorax incidence [13]. Whether oxygen administered during the biopsy before pneumothorax detection is associated with postprocedural pneumothorax remains unclear.

The primary aim of this study was to evaluate whether documented intraprocedural supplemental oxygen administration was associated with postprocedural pneumothorax in patients undergoing CT-guided TLB. The secondary aim was to assess clinical, imaging, and procedural factors associated with pneumothorax.

## Methods

### Study design and patient population

This single-center retrospective cohort study was conducted in the Department of Radiology at Marmara University. Consecutive patients who underwent CT-guided TLB for pulmonary lesions between January 2022 and June 2025 were retrospectively evaluated. For patients who underwent more than one biopsy during the study period, only the first eligible procedure was included. Preprocedural CT images, biopsy procedure records, hospital information system data, oxygen administration records, postprocedural imaging findings, and pathology reports were reviewed.

**Fig. 1:**
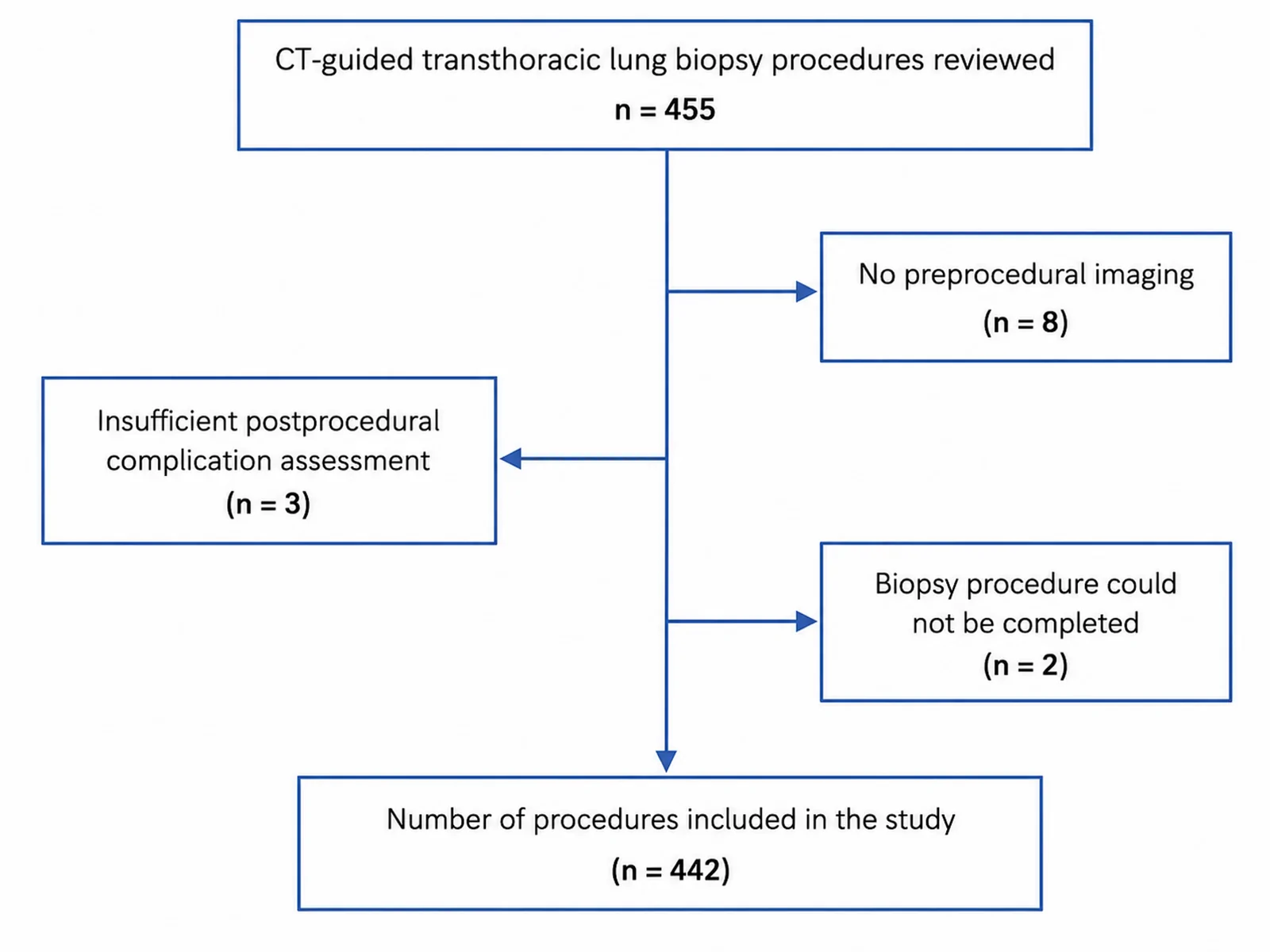
Flow chart showing selection of CT-guided transthoracic lung biopsy procedures included in the study

A total of 455 potentially eligible biopsy procedures were reviewed. Procedures were excluded because preprocedural CT images were unavailable (*n* = 8), archived immediate postprocedural CT images were incomplete or inaccessible (*n* = 3), or the biopsy could not be completed (*n* = 2). The final study population consisted of 442 patients, each contributing one biopsy procedure. Assessable immediate postprocedural CT images were available for all patients included in the final cohort. The patient selection process is shown in Fig. 1.

### Clinical variables

Age, sex, diabetes mellitus, hypertension, cardiovascular disease, chronic obstructive pulmonary disease, and history of extrapulmonary malignancy were recorded. Chronic kidney disease, chronic liver disease, neurological disorders, and rheumatological diseases were grouped as other comorbidities.

Biopsy results were categorized as malignant or benign based on the final histopathological diagnosis. Primary lung malignancies, metastatic tumors, lymphoproliferative disorders, and other malignant neoplastic infiltrations were classified as malignant. Inflammatory, granulomatous, organizing pneumonia, fibrotic, hyalinized, reactive, and other non-neoplastic findings were classified as benign. No biopsy yielded a nondiagnostic or indeterminate histopathological result in the final study cohort.

### CT-guided transthoracic lung biopsy procedure

All biopsies were performed using a coaxial core-biopsy technique under sequential CT guidance; CT fluoroscopy was not used. Patients were instructed to briefly hold their breath during needle advancement and CT image acquisition. Patient position was selected according to lesion location and the planned access route and was recorded as prone, supine, or lateral decubitus. After local anesthesia, tissue samples were obtained using an 18-gauge core biopsy needle through a 17-gauge coaxial introducer. All procedures were performed by a single attending radiologist with 8 years of radiology experience, including 5 years in interventional radiology. No major change in the biopsy technique or routine oxygen-administration practice occurred during the study period.

Biopsy duration was recorded in minutes and defined as the interval between the initial needle insertion and removal of the coaxial introducer needle. Immediate postprocedural CT images were available for all patients included in the final study cohort to assess procedure-related complications.

### Assessment of intraprocedural oxygen administration

Intraprocedural supplemental oxygen administration was determined from the biopsy procedure notes, in which oxygen use was routinely recorded. Patients with a record of oxygen administration during the biopsy and before pneumothorax detection were assigned to the oxygen group. Patients with no record of oxygen administration during this period were assigned to the no-oxygen group. Because an independent source was not consistently available for validation, occasional exposure misclassification due to undocumented oxygen administration cannot be excluded. Oxygen initiated only after pneumothorax detection as part of clinical management was not considered intraprocedural oxygen exposure.

Oxygen administration was part of routine clinical care and was not based on a standardized study protocol. The decision to administer oxygen was made by the procedural team according to clinical need. Oxygen was generally initiated before the biopsy and discontinued when the procedure was completed. Oxygen initiated during the procedure before radiological detection of pneumothorax was also classified as exposure. It was generally delivered at 4–6 L/min using a nasal cannula in the prone position and either a nasal cannula or face mask in the supine or lateral decubitus position. The records allowed us to determine whether oxygen had been administered before pneumothorax detection; however, the exact initiation time, clinical indication, oxygen concentration, duration, and patient-specific flow rate and delivery method could not be consistently verified. Therefore, documented oxygen administration was considered a non-standardized clinical exposure and analyzed as a binary variable.

### CT image analysis and radiological variables

All imaging measurements and classifications were performed by a single radiologist specifically for the present study. The evaluated variables included lesion size, cavitation, CT-detected emphysema, pleural distance, lesion–pleura relationship, subcutaneous tissue thickness, and needle angle.

Lesion size was assessed on the axial image showing the largest lesion area by measuring the maximum diameter and the perpendicular short-axis diameter in millimeters. A cavitary lesion was defined as a lesion containing an air-filled cavitary component.

Pleural distance was measured along the biopsy trajectory from the pleural entry point to the nearest margin of the target lesion. Lesions directly contacting the pleura, corresponding to a pleural distance of 0 mm, were classified as pleural-based. Lesions separated from the pleura by any intervening aerated lung parenchyma, corresponding to a pleural distance greater than 0 mm, were classified as non-pleural-based.

**Fig. 2:**
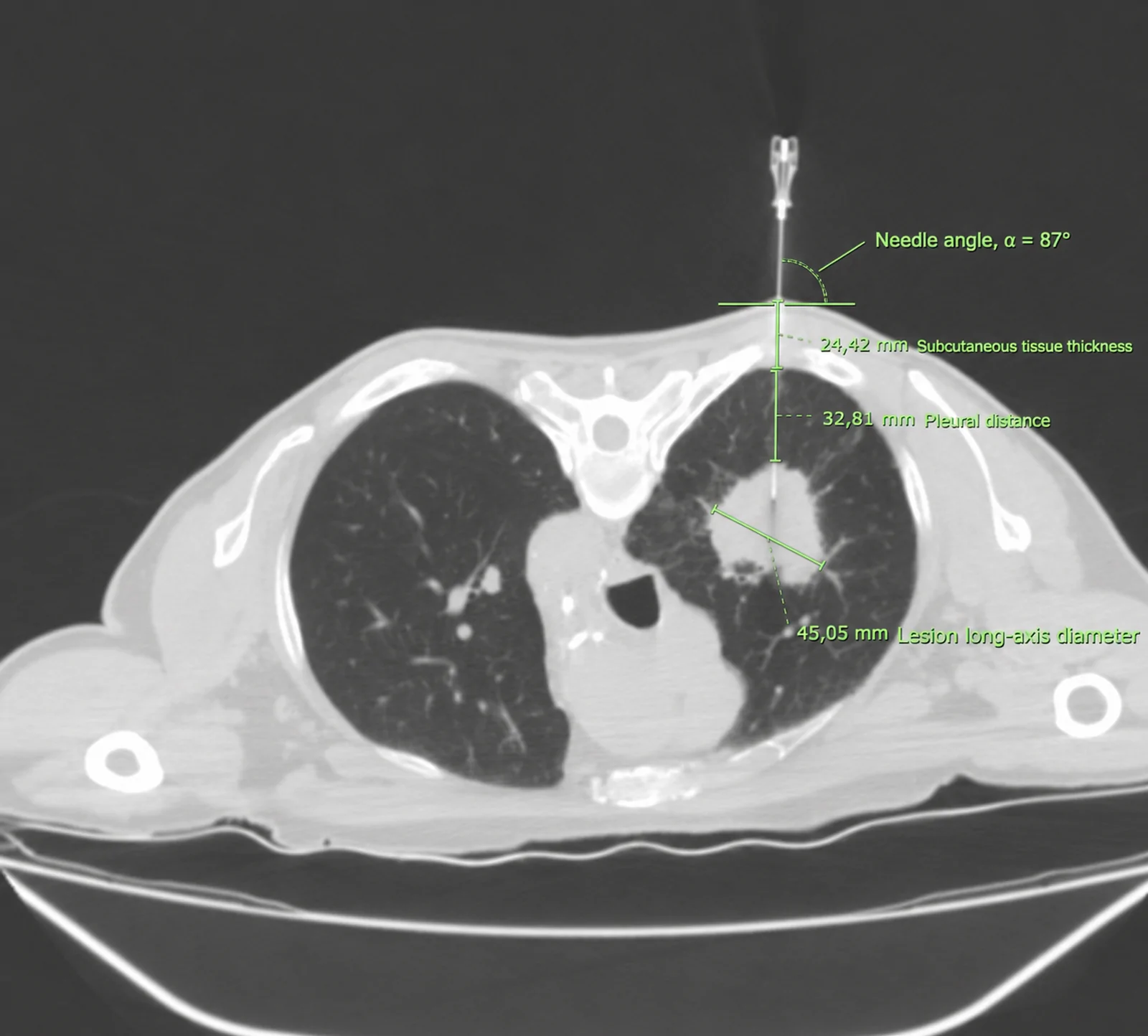
Axial CT image during CT-guided transthoracic lung biopsy demonstrating measurement of lesion size, pleural distance, subcutaneous tissue thickness, and needle angle

Subcutaneous tissue thickness was measured along the biopsy route from the skin entry point to the pleural entry point. Needle angle was defined as the acute angle between the needle trajectory and the tangent to the pleural surface at the puncture site, ranging from 0° to 90°. CT-detected emphysema was defined as visually identifiable emphysematous change in the ipsilateral lung, including along or adjacent to the needle trajectory. The measurement methods are illustrated in Fig. 2.

### Outcomes

**Fig. 3:**
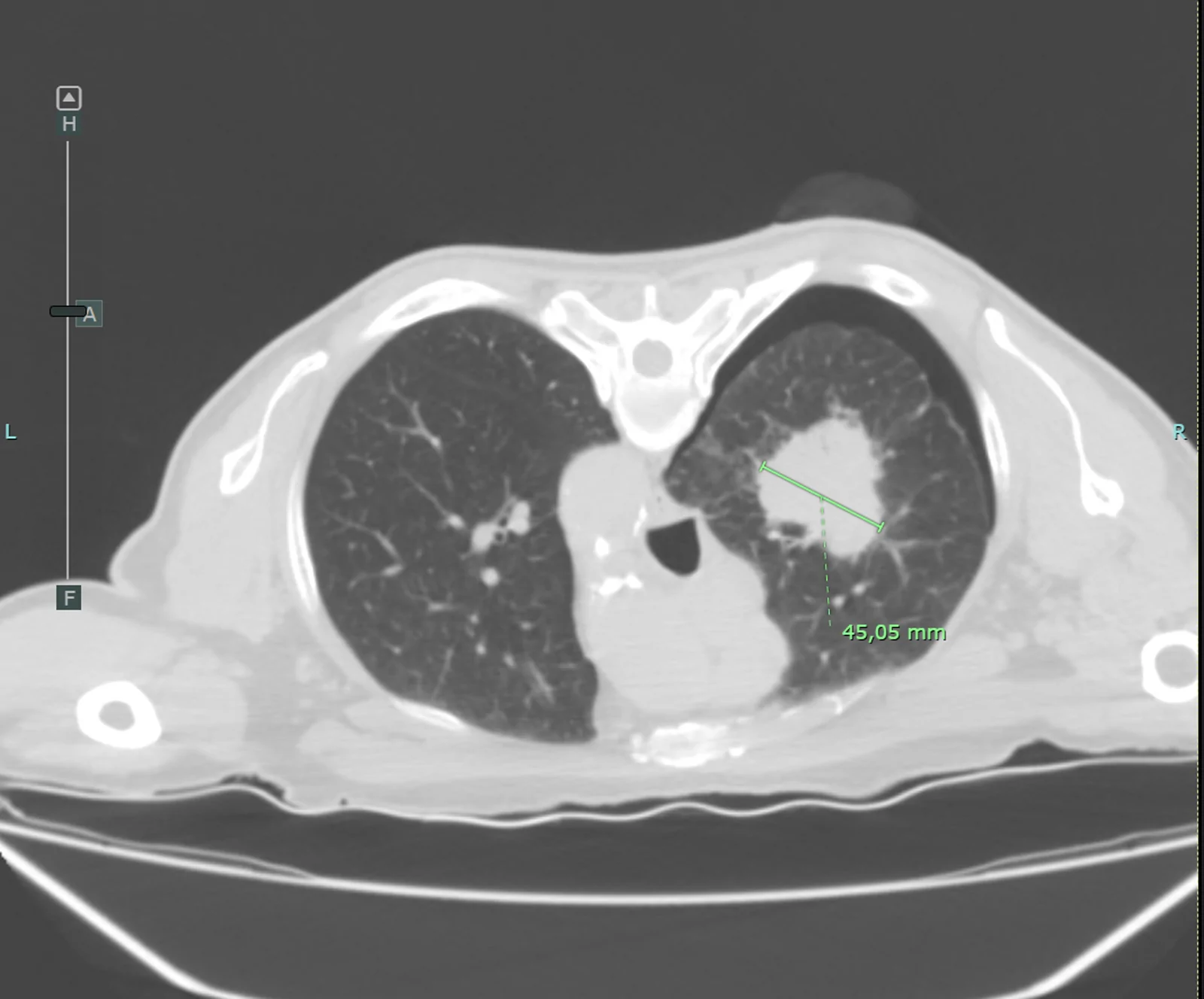
Axial CT image after CT-guided transthoracic lung biopsy demonstrating postprocedural pneumothorax adjacent to the biopsied lesion

The primary outcome was postprocedural pneumothorax. Pneumothorax was primarily assessed on immediate postprocedural CT in all patients. Patients were subsequently observed for 4–6 h, after which follow-up posteroanterior chest radiography was obtained. Pneumothoraces detected on either immediate CT or follow-up chest radiography were included in the analysis. A representative case is shown in Fig. 3.

Secondary outcomes were pulmonary hemorrhage and pneumothorax-related intervention. Pulmonary hemorrhage was defined as newly developed ground-glass opacity or consolidation along the needle tract or surrounding the target lesion on postprocedural imaging.

Pneumothorax-related intervention was defined as air aspiration, pleural catheter placement, or chest tube insertion for management of post-biopsy pneumothorax. Intervention was evaluated both in the entire cohort and separately among patients who developed pneumothorax. Because pneumothorax size was not directly measured, the need for intervention was evaluated as a pragmatic indicator of clinical significance rather than a direct measure of pneumothorax size or severity.

### Statistical analysis

Statistical analyses were performed using SPSS Statistics, version 27.0 (IBM Corp., Armonk, NY, USA). Continuous variables were summarized as medians with interquartile ranges and compared between the oxygen and no-oxygen groups using the Mann–Whitney U test. Categorical variables were presented as frequencies and percentages and compared using the chi-square test. For continuous variables, ASDs were calculated as the difference in group means divided by the square root of the average of the two group variances. For binary variables, ASDs were calculated as the difference in proportions divided by the square root of the average of the two group-specific binomial variances; generalized ASDs were used for variables with more than two categories.

Absolute risk differences and their 95% confidence intervals were estimated using generalized linear models with a binomial distribution and identity link. Risk differences were calculated as the event rate in the oxygen group minus that in the no-oxygen group.

Univariable logistic regression analyses were performed to assess the unadjusted association of each prespecified variable with pneumothorax. Variables included in the multivariable model were selected a priori based on clinical relevance and previously reported associations with pneumothorax, rather than on univariable statistical significance alone. Oxygen administration, age, sex, CT-detected emphysema, lesion long-axis diameter, lesion–pleura relationship, needle angle, subcutaneous tissue thickness, and patient position were entered simultaneously into the multivariable model. Patient position was included as a categorical variable, with the prone position as the reference category. Pleural distance and lesion–pleura relationship were not included in the same model because of conceptual overlap. Similarly, chronic obstructive pulmonary disease and CT-detected emphysema were not entered together; CT-detected emphysema was selected because it more directly reflected biopsy-related parenchymal risk.

Linearity in the logit of continuous predictors was assessed using Box–Tidwell tests with Bonferroni correction for four tests (adjusted significance level, 0.0125), and multicollinearity was evaluated using tolerance and variance inflation factor values. Model discrimination, calibration, and overall prediction error were assessed using the area under the receiver operating characteristic curve with a nonparametric 95% confidence interval, the Hosmer–Lemeshow goodness-of-fit test, and the Brier score, respectively. Results of the logistic regression analyses were reported as odds ratios with 95% confidence intervals. All 442 patients had complete data for the variables included in the multivariable model. No formal sample-size calculation was performed because all consecutive eligible patients undergoing CT-guided TLB during the study period were included. A p value < 0.05 was considered statistically significant, except for the Box–Tidwell tests, for which the Bonferroni-adjusted significance level was 0.0125.

## Results

A total of 442 consecutive patients undergoing CT-guided TLB were included, with one procedure evaluated per patient. Intraprocedural supplemental oxygen administration was documented in 118 patients (26.7%), whereas no intraprocedural oxygen administration was documented in 324 patients (73.3%).

Baseline clinical characteristics are presented in Table 1. Median age was slightly lower in the oxygen group than in the no-oxygen group [66 years (56–72) vs. 67 years (61–74); *p* = 0.026]. No statistically significant between-group differences were observed in the other measured clinical characteristics, including sex, diabetes mellitus, hypertension, cardiovascular disease, chronic obstructive pulmonary disease, other comorbidities, history of extrapulmonary malignancy, and biopsy results.

**Table 1:** Clinical characteristics and biopsy results according to oxygen administration

Variable	Oxygen group (*n* = 118)	No-oxygen group (*n* = 324)	ASD	*p* value
Age (years)	66 (56–72)	67 (61–74)	0.287	**0.026**
Male sex, n (%)	79 (66.9)	236 (72.8)	0.129	0.226
Diabetes mellitus, n (%)	23 (19.5)	54 (16.7)	0.073	0.489
Hypertension, n (%)	35 (29.7)	72 (22.2)	0.170	0.106
Cardiovascular disease, n (%)	24 (20.3)	55 (17.0)	0.086	0.414
COPD, n (%)	16 (13.6)	37 (11.4)	0.065	0.540
Other comorbidity, n (%)	15 (12.7)	26 (8.0)	0.154	0.133
Extrapulmonary malignancy, n (%)	31 (26.3)	66 (20.4)	0.140	0.185
Biopsy result, n (%) Malignant Benign	74 (62.7) 44 (37.3)	227 (70.1) 97 (29.9)	0.156	0.142

Values are presented as median (interquartile range) or n (%). Other comorbidity included rheumatologic, neurologic, hepatic, or renal disease

Abbreviations: ASD, absolute standardized difference; COPD, chronic obstructive pulmonary disease

Lesion and procedural characteristics are summarized in Table 2. Compared with the no-oxygen group, procedures in the oxygen group had a lower median needle angle [77° (61–83) vs. 80° (65–85); *p* = 0.034], a longer median biopsy duration [6 min (5–7) vs. 5 min (4–7); *p* = 0.001], and greater median subcutaneous tissue thickness [38 mm (29–45) vs. 33 mm (25–41); *p* = 0.007]. No statistically significant between-group differences were observed in lesion size, cavitation, CT-detected emphysema, pleural distance, lesion–pleura relationship, or patient position.

**Table 2:** Lesion and procedural characteristics according to oxygen administration

Variable	Oxygen group (*n* = 118)	No-oxygen group (*n* = 324)	ASD	*p* value
Lesion size, long-axis diameter (mm)	33 (19–49)	34 (22–52)	0.065	0.368
Lesion size, short-axis diameter (mm)	23 (15–36)	24 (16–38)	0.015	0.699
Cavitary lesion, n (%)	14 (11.9)	30 (9.3)	0.085	0.418
CT-detected emphysema, n (%)	10 (8.5)	25 (7.7)	0.028	0.794
Needle angle (degrees)	77 (61–83)	80 (65–85)	0.175	**0.034**
Biopsy duration (minutes)	6 (5–7)	5 (4–7)	0.194	**0.001**
Subcutaneous tissue thickness (mm)	38 (29–45)	33 (25–41)	0.296	**0.007**
Pleural distance (mm)	10 (0–23)	8 (0–21)	0.142	0.203
Lesion-pleura relationship, n (%) Pleural-based Non–pleural-based	39 (33.1) 79 (66.9)	123 (38.0) 201 (62.0)	0.103	0.343
Patient position, n (%) Prone Supine Lateral decubitus	61 (51.7) 26 (22.0) 31 (26.3)	191 (59.0) 80 (24.7) 53 (16.4)	0.244	0.063

Values are presented as median (interquartile range) or n (%)

Abbreviations: ASD, absolute standardized difference; CT, computed tomography

Procedure-related outcomes are presented in Table 3. Pneumothorax occurred in 141 patients (31.9%). It developed in 36 of 118 patients in the oxygen group (30.5%) and 105 of 324 patients in the no-oxygen group (32.4%), corresponding to an absolute risk difference of − 1.9% points (95% CI, − 11.6 to 7.8; *p* = 0.705). Among the 35 patients who required pneumothorax-related intervention, 5 (14.3%) underwent air aspiration, 14 (40.0%) underwent pleural catheter placement, and 16 (45.7%) underwent chest tube insertion. Pulmonary hemorrhage occurred in 53 of 118 patients in the oxygen group (44.9%) and 127 of 324 patients in the no-oxygen group (39.2%), corresponding to an absolute risk difference of 5.7% points (95% CI, − 4.7 to 16.1; *p* = 0.279). Pneumothorax-related intervention was required in 11 of 118 patients in the oxygen group (9.3%) and 24 of 324 patients in the no-oxygen group (7.4%), corresponding to an absolute risk difference of 1.9% points (95% CI, − 4.1 to 7.9; *p* = 0.510). Among patients who developed pneumothorax, pneumothorax-related intervention was required in 11 of 36 patients in the oxygen group (30.6%) and 24 of 105 patients in the no-oxygen group (22.9%), corresponding to an absolute risk difference of 7.7% points (95% CI, − 9.4 to 24.8; *p* = 0.356).

**Table 3:** Procedure-related outcomes according to intraprocedural oxygen administration

Variable	Oxygen group (*n* = 118)	No-oxygen group (*n* = 324)		*p* value
Pneumothorax, n (%)	36 (30.5)	105 (32.4)	−1.9 (− 11.6 to 7.8)	0.705
Pulmonary hemorrhage, n (%)	53 (44.9)	127 (39.2)	5.7 (− 4.7 to 16.1)	0.279
Pneumothorax-related intervention, n (%)	11 (9.3)	24 (7.4)	1.9 (− 4.1 to 7.9)	0.510
Pneumothorax-related intervention among patients with pneumothorax, n/N (%)	11/36 (30.6)	24/105 (22.9)	7.7 (− 9.4 to 24.8)	0.356

Values are presented as n (%) unless otherwise indicated. Pneumothorax-related intervention included air aspiration, pleural catheter placement, or chest tube insertion. Risk differences are expressed in percentage points and were calculated as the event rate in the oxygen group minus that in the no-oxygen group.

The results of the univariable and multivariable logistic regression analyses are presented in Table 4. Intraprocedural oxygen administration was not significantly associated with pneumothorax in the univariable analysis (OR, 0.916; 95% CI, 0.581–1.444; *p* = 0.705) or after multivariable adjustment (adjusted OR, 0.807; 95% CI, 0.492–1.323; *p* = 0.395). In the multivariable analysis, non-pleural-based lesion location was associated with higher odds of pneumothorax (adjusted OR, 3.060; 95% CI, 1.852–5.054; *p* < 0.001), as was CT-detected emphysema (adjusted OR, 2.179; 95% CI, 1.009–4.705; *p* = 0.047). Greater subcutaneous tissue thickness was associated with lower odds of pneumothorax (adjusted OR per 1 mm increase, 0.973; 95% CI, 0.956–0.991; *p* = 0.003). Patient position was also associated with pneumothorax overall (*p* = 0.003). Compared with the prone position, the lateral decubitus position was associated with higher odds of pneumothorax (adjusted OR, 2.448; 95% CI, 1.403–4.269; *p* = 0.002), whereas the supine position was not significantly associated with pneumothorax (adjusted OR, 0.924; 95% CI, 0.538–1.587; *p* = 0.775). All continuous predictors satisfied the linearity-in-the-logit assumption according to the Bonferroni-adjusted Box–Tidwell tests (all *p* ≥ 0.029; adjusted significance level, 0.0125). No relevant multicollinearity was detected (minimum tolerance, 0.857; maximum variance inflation factor, 1.166). The model showed moderate discrimination (AUC, 0.701; 95% CI, 0.650–0.751). The Hosmer–Lemeshow test did not indicate lack of fit (χ² = 11.867, df = 8, *p* = 0.157), and the Brier score was 0.194.

**Table 4:** Univariable and multivariable logistic regression analyses of factors associated with pneumothorax

Variable	Univariable		Multivariable	
	Univariable OR (95% CI)	*p* value	Multivariable OR (95% CI)	*p* value
Oxygen administration	0.916 (0.581–1.444)	0.705	0.807 (0.492–1.323)	0.395
Age (years)	0.999 (0.980–1.018)	0.922	0.990 (0.970–1.010)	0.313
Male sex	1.199 (0.765–1.879)	0.428	1.162 (0.705–1.916)	0.555
Lesion size, long-axis diameter (mm)	0.993 (0.983–1.002)	0.143	0.998 (0.987–1.009)	0.700
CT-detected emphysema	2.155 (1.075–4.322)	**0.030**	2.179 (1.009–4.705)	**0.047**
Non–pleural-based lesion	3.057 (1.916–4.879)	**< 0.001**	3.060 (1.852–5.054)	**< 0.001**
Needle angle (degrees)	0.990 (0.979–1.001)	**0.063**	0.991 (0.979–1.003)	0.128
Subcutaneous tissue thickness (mm)	0.982 (0.966–0.998)	**0.027**	0.973 (0.956–0.991)	**0.003**
Patient position Prone Supine Lateral decubitus	Reference 0.863 (0.519–1.438) 2.085 (1.255–3.462)	**0.006** **—** 0.573 **0.005**	Reference 0.924 (0.538–1.587) 2.448 (1.403–4.269)	**0.003** **—** 0.775 **0.002**

Values are presented as odds ratios with 95% confidence intervals (CIs). Odds ratios for continuous predictors are expressed per 1-unit increase. The multivariable model included all variables shown and was based on 442 patients with 141 pneumothorax events. The prone position was the reference category. The overall p value for patient position represents the joint test across categories; category-specific p values compare the supine and lateral decubitus positions with the prone position. Abbreviation: CT, computed tomography

## Discussion

In this single-center retrospective cohort study, documented intraprocedural oxygen administration was not significantly associated with postprocedural pneumothorax after CT-guided TLB. Non-pleural-based lesion location, CT-detected emphysema, and lower subcutaneous tissue thickness were associated with pneumothorax.

The pneumothorax rate in our cohort was somewhat higher than the pooled estimate reported by Huo et al., but remained within the range described in previous large studies and meta-analyses [3, 4, 7]. Huo et al. reported pooled rates of 25.9% for pneumothorax and 6.9% for chest drain insertion [4]. In our cohort, pneumothorax occurred in 31.9% of patients, and 7.9% required an intervention. Although the intervention rates were similar, direct comparison should be made cautiously because our definition also included air aspiration and pleural catheter placement. The higher overall pneumothorax rate in our study may partly reflect the use of immediate postprocedural CT in all patients, which allowed the detection of small, clinically silent pneumothoraces that might not have been identified in studies relying mainly on chest radiography or symptom-triggered imaging.

Our findings regarding oxygen administration should be interpreted with caution. Supplemental oxygen has traditionally been used in the management of pneumothorax to facilitate pleural air resorption, and previous studies suggest that oxygen may accelerate radiographic resolution in selected patients with spontaneous pneumothorax [10, 11]. This effect is thought to occur through nitrogen washout, which increases the diffusion of nitrogen from the pleural space into the blood and accelerates pleural-air resorption [11]. Biopsy-specific evidence is limited and inconsistent. In an early fluoroscopy-guided study, 100% oxygen administered from 5 min before transthoracic needle biopsy until 30 min after the procedure was associated with fewer and smaller pneumothoraces [12]. In contrast, Poe et al. reported that 100% oxygen during fluoroscopy-guided TLB did not significantly reduce pneumothorax incidence, although it may have reduced pneumothorax size [13]. In both studies, pneumothorax was assessed using chest radiography, whereas our CT-based assessment may have detected smaller or earlier pleural air collections. This difference in outcome ascertainment and detection timing further limits direct comparison with their findings. A recent review similarly emphasized that the supporting clinical evidence remains limited and methodologically heterogeneous [11]. Unlike these historical TLB studies using protocolized 100% oxygen, our study evaluated clinically indicated, non-standardized oxygen administration during CT-guided TLB using a coaxial core-biopsy technique. Oxygen was generally delivered at 4–6 L/min via a nasal cannula or face mask, and patient-level FiO₂, timing, and duration could not be consistently verified. Moreover, oxygen would not be expected to mechanically prevent visceral pleural injury or air entry along the needle tract. Thus, any effect on established pleural air would represent a therapeutic effect rather than prevention of the initial pleural injury. Because oxygen was generally administered only during the brief biopsy period, our study did not evaluate the sustained oxygen exposure intended to accelerate the resorption of established pleural air. Oxygen administration to maintain oxygenation or improve procedural tolerance represents a separate clinical indication that was not evaluated in this study. Therefore, our findings do not exclude a potential effect of standardized high-concentration oxygen on pleural-air resorption; rather, no statistically significant association was identified between documented intraprocedural oxygen administration and postprocedural pneumothorax.

At the same time, the absence of an observed association should not be taken to mean that oxygen has no clinical benefit. Because oxygen was administered according to clinical need rather than a standardized protocol, patients who received oxygen may have differed from those who did not in terms of oxygenation, respiratory symptoms, breath-holding ability, or procedural tolerance. Among patients who developed pneumothorax, intervention was numerically more frequent in the oxygen group, although the difference was not statistically significant. Because oxygen exposure was defined before pneumothorax detection, this finding should not be interpreted as evidence that oxygen increased the need for intervention. Instead, it may reflect unmeasured differences in clinical status or procedural difficulty between the groups. Accordingly, our findings should be interpreted as an exploratory observational analysis of the association between documented, non-standardized intraprocedural oxygen administration and postprocedural pneumothorax, rather than as an assessment of the efficacy of a standardized oxygen intervention.

The factors associated with pneumothorax in our study were largely consistent with previous reports [3–7]. Li et al. identified CT-detected emphysema as an independent predictor of pneumothorax [5], whereas Deng et al. reported that the nodule–pleura relationship and the depth of intrapulmonary needle insertion influenced pneumothorax risk [7]. Consistent with these findings, non-pleural-based lesion location and CT-detected emphysema were independently associated with pneumothorax in our cohort. Previous meta-analyses have similarly shown that emphysema, lack of pleural contact, and a longer needle path through aerated lung increase the risk of pneumothorax after CT-guided lung biopsy [3, 4]. The association with non-pleural-based lesions is clinically plausible because reaching these lesions requires the needle to pass through aerated lung parenchyma. This also supports the importance of minimizing the intrapulmonary needle trajectory and selecting the shortest feasible biopsy route. Other technical factors, including multiple pleural punctures and crossing a fissure or bulla, have also been associated with an increased risk of pneumothorax [4]; however, these variables were not consistently available in our study. The higher risk observed with lateral decubitus positioning should be interpreted cautiously because our analysis did not distinguish between biopsy-side-down and biopsy-side-up positioning, and patient position was selected according to lesion location and the planned access route. The meta-analysis by Huo et al. reported a lower pneumothorax risk when the biopsied lung was dependent and a higher risk when it was non-dependent [4]. Drumm et al. similarly reported fewer pneumothoraces with biopsy-side-down positioning than with prone or supine positioning [14]. Therefore, our finding may reflect patient orientation, case selection, or procedural complexity rather than an adverse effect of lateral positioning itself. The inverse association between greater subcutaneous tissue thickness and pneumothorax was unexpected and should also be interpreted cautiously. It may reflect differences in body habitus or other unmeasured procedural characteristics rather than a direct protective effect. Because body mass index and other anthropometric measures were unavailable, this possibility could not be further evaluated. Therefore, this finding should be considered exploratory.

The rate of pulmonary hemorrhage did not differ significantly between the oxygen and no-oxygen groups. In our study, pulmonary hemorrhage was defined as newly developed ground-glass opacity or consolidation along the needle tract or around the target lesion on postprocedural imaging. This imaging-based definition may explain the relatively high hemorrhage rate compared with studies that considered only clinically evident hemoptysis. Reported hemorrhage rates vary according to the definition used, the timing of postprocedural imaging, and the grading method [15–18]. De Filippo et al. reported that pulmonary hemorrhage is common after CT-guided TLB and that more extensive needle-tract hemorrhage may be associated with a lower incidence of pneumothorax [18]. In the present study, however, no significant association was observed between documented intraprocedural oxygen administration and pulmonary hemorrhage.

This study has several limitations. First, it was retrospective and conducted at a single center. Because of the observational design, the identified associations should not be interpreted as causal relationships. The number of pleural punctures and other potentially relevant procedural details, including the number of core samples, needle repositioning, crossing of a fissure, bulla, or emphysematous region, and intrapulmonary needle-trajectory length, were not consistently available and could not be included in the analysis. This may have resulted in residual procedural confounding. In addition, all imaging measurements were performed by a single radiologist, and intraobserver and interobserver reproducibility was not assessed. Anthropometric measures such as body mass index were not available, limiting further assessment of whether body habitus contributed to the inverse association between subcutaneous tissue thickness and pneumothorax. Second, oxygen administration was evaluated only as a documented binary exposure. Patient-specific clinical indication, exact initiation time, its relationship to needle insertion or pleural puncture, duration, oxygen concentration, flow rate, and delivery method could not be consistently verified, precluding reliable reporting of their distributions. This classification combined heterogeneous forms of oxygen administration and may have obscured associations related to dose, timing, or delivery method. Because patients without documented oxygen administration were classified as the no-oxygen group and independent validation against nursing or monitoring records was not consistently possible, some exposure misclassification cannot be excluded. Although oxygen was generally started before biopsy, intraprocedural use before radiological detection was also classified as exposure; because exact timing relative to pleural puncture and pneumothorax onset was unavailable, a pre-puncture sensitivity analysis was not feasible and reverse causation cannot be excluded. Potential confounders and clinical determinants of oxygen administration including baseline and intraprocedural oxygen saturation, pre-existing oxygen requirement, intraprocedural dyspnea, respiratory rate, coughing, anxiety, breath-holding capacity, patient movement and the specific reason for oxygen administration were not consistently available. Because these variables may have influenced both oxygen administration and pneumothorax risk, confounding by indication is a central limitation and could not be adequately addressed by multivariable adjustment. Accordingly, the findings should not be interpreted as an evaluation of a standardized oxygen intervention. Pneumothorax size, exact time to detection, timing of intervention, time to resolution, and subsequent clinical course were not consistently available. Although the need for intervention was assessed and intervention types were reported, this did not directly quantify pneumothorax size or severity. Therefore, the potential effect of oxygen on pleural-air accumulation or resolution could not be evaluated.

## Conclusion

No statistically significant independent association was identified between documented intraprocedural oxygen administration and postprocedural pneumothorax after CT-guided TLB. However, the wide confidence intervals remained compatible with both clinically relevant benefit and harm. Given the retrospective design, non-standardized oxygen administration, and unavailable determinants of oxygen use, these findings should not be interpreted as evidence of no effect. Among the other evaluated factors, non-pleural-based lesion location and CT-detected emphysema were associated with higher odds of pneumothorax. Greater subcutaneous tissue thickness was associated with lower odds, but this unexpected finding should be considered exploratory. Prospective studies using standardized oxygen protocols are needed to determine whether a preventive effect exists.

## Data Availability

The datasets generated and analyzed during the current study are available from the corresponding author on reasonable request.

## Abbreviations

CI: Confidence interval
CT: Computed tomography
OR: Odds ratio
TLB: Transthoracic lung biopsy
